# Awareness, understanding and use of sodium information labelled on pre-packaged food in Beijing:a cross-sectional study

**DOI:** 10.1186/s12889-018-5396-7

**Published:** 2018-04-17

**Authors:** Yao He, Liping Huang, Sijin Yan, Yuan Li, Lixin Lu, Hongbo Wang, Wenyi Niu, Puhong Zhang

**Affiliations:** 1 The George Institute for Global Health at Peking University Health Science Center, Level 18, Tower B, Horizon Tower, No. 6, Zhichun Road, Haidian District, Beijing, 100088 China; 2 Department of Social Medicine and Health education, School of Public Health, Peking University, No. 38 Xueyuan Road, Haidian District, Beijing, 100191 China; 3 Xicheng District Center for Disease Control and Prevention, No. 38, Waidajie, Deshengmen, Xicheng District, Beijing, 100120 China; 4 Haidian District Administration Center for Community Health Service, No. 12, Ganjiakou community, Haidian District, Beijing, China

**Keywords:** Sodium, Salt, Nutrition labelling, Awareness, Understanding, Use

## Abstract

**Background:**

Nutrition labelling has been mandatory for pre-packaged foods since 2013 in China, and sodium is one of the nutrients required for display on the nutritional information panel (NIP). This study aimed to estimate the awareness, understanding of, and use of sodium labelling information among the population in China.

**Methods:**

A cross-sectional survey was carried out in urban Beijing in 2016 on pre-packaged foods. The researchers randomly selected 380 residents from four convenient but disconnected communities and 370 shoppers from four supermarkets owned by different companies and conducted face-to-face interviews. Questions on nutritional knowledge, health attitude, understanding and use of nutritional labels as well as other related factors were assessed.

**Results:**

All of the 380 community residents and 308 of the 370 supermarket shoppers successfully completed the survey. Of those 688 respondents, 91.3% understood that excessive salt intake was harmful, 19.5% were aware that sodium content is listed on the NIP, 5.5% understood the meaning of NRV% (Percentage of Nutrient Reference Values), 47.7% did not know the relationship between sodium and salt, and 12.6% reported they frequently read the label when shopping. Factors for why people were more likely to choose a product because of its low level of salt shown on the label include income level and their level of awareness of the link between salt and diet.

**Conclusions:**

Although the participants had a good understanding of the harmful effects of salt, the awareness, understanding and use of sodium labels was very low in Beijing, and even worse nationwide. Efforts should be taken to educate the public to understand and use the NIP better and design clearer ways of displaying such information, such as front-of pack (FoP) labelling or health-related smartphone applications to improve health and help people make better food choices.

**Electronic supplementary material:**

The online version of this article (doi:10.1186/s12889-018-5396-7) contains supplementary material, which is available to authorized users.

## Background

It is well documented that there is a strong relationship between excessive sodium intake and hypertension [1–4]. Sodium differs from salt (sodium chloride). But for the purpose of this paper, we use “sodium” to mean “salt”. The implementation of effective salt reduction strategies has been regarded as one of the most cost-effective measures to prevent cardiovascular diseases [5–7]. Member States of the World Health Organization (WHO) have agreed to a global target of a 30% reduction in population salt intake by 2025 [8]. Salt intake in China is known to be higher than in most other countries [9, 10], two or three times higher than the 5 g daily limit recommended by WHO [11]. Hence, effective salt reduction programs are urgently needed in China.

Pre-packaged processed foods are the major source of salt intake in developed countries and their consumption is rising in many developing countries [10]. Some governments have initiated national approaches to work with the food industry to reduce salt in foods by setting salt reduction targets [12–14]. Meanwhile, labelling the amount of sodium and/or salt on the nutrition information panel (NIP) of pre-packaged foods is also among health initiatives to reduce salt. Many countries have added sodium or salt to the list of required nutrients that must be declared on the NIP [15–19]. Some countries, like the US, now highlight the sodium content of food in larger, bold font in black on the label, to draw consumers’ attention to the sodium content in pre-packaged food. Although pre-packaged food is not the main source of salt intake in China, the sales and consumption of pre-packaged food are increasing very quickly and becoming an important source of salt intake.

**Fig. 1:**
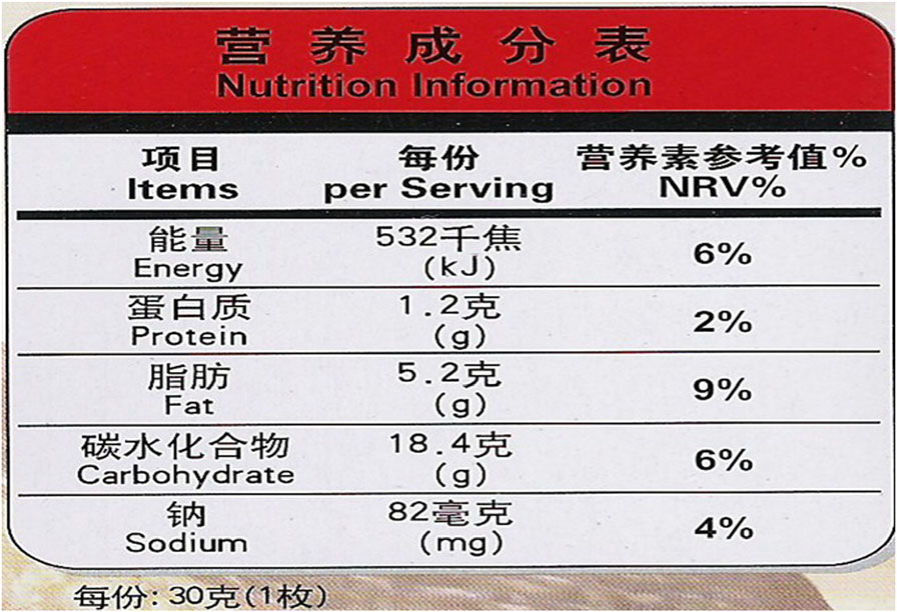
A sample nutrition information panel for pre-packaged food in China

The Chinese Government introduced nutrition labelling regulations (General Rules for Nutrition Labelling of Pre-packaged foods - GB 28050–2011) in 2011 and officially implemented the regulation from January 1, 2013 (Fig. 1). These regulations require all pre-packaged food, with some exceptions (some fresh foods, alcohol, etc.) to display the energy content (kJ), as well as the amount of protein (g), total fat (g), carbohydrate (g) and sodium (mg) on the NIP. The nutritional values are displayed per 100 g, or per 100 ml or per serving of the food. The sodium label or labelling, in this study, means the sodium concentration shown in mg per 100 g or per 100 ml or per serving on the NIP, as well as its nutrient reference value (NRV%).

To our knowledge, few studies have evaluated the awareness, understanding and use of current sodium labelling among Chinese consumers. This cross-sectional study tries to answer these questions and explore the factors associated with them. In this paper, we also recommend changes and improvements to the current sodium labelling system.

## Methods

### Study design and population

A cross-sectional survey was conducted in Beijing from April to May 2016. Participants were chosen from four convenient but disconnected communities and four supermarkets owned by different companies, with the purpose of representing subgroups with different understanding and use of sodium label due to different ages, education, economic level and personal preference of purchasing and consuming pre-packaged food. Those interviewed in communities were usually older and many retired, with lower incomes and more health problems, while the shoppers at supermarkets were on average, younger in better health and were earning a higher income. In each community, households were visited to find at least 80 adult participants. Only one family member was invited to participate in the survey. If there were two or more family members willing to participate, the person with a higher level of education was selected. In each supermarket, 80 adult shoppers were invited at the entrance to the store to take part in the study. All the participants were interviewed face to face and a standard questionnaire was filled out. A ‘non-respondent’ was defined as someone who chose not to participate in the survey. People under 18 years old or unable to read for any reason were excluded from participating in the survey.

### Data collection

Self-completed paper-based questionnaires were distributed to all study participants on each site. Participants were requested to complete the questionnaire anonymously and independently after the investigators explained how to fill in the questionnaire. Incomplete questionnaires were excluded from the analysis. Some shoppers declined to participate or to answer all the questions, mainly due to a lack of time to participate.

### Questionnaire design and definition

The questionnaires were designed to collect the following information: **1)** Social demographic characteristics, including age, sex, height, weight, education, and household monthly income per capita; **2)** Self-reported existing health problems, such as diabetes, hypertension, cardiovascular diseases, dyslipidemia, fatty liver, chronic kidney disease, and food allergies; **3)** Awareness of sodium labelling on food packaging, i.e. knowing that “sodium” must be labelled on the NIP as the requirement of government; **4)** Understanding of the relationship between sodium and salt, i.e. knowing “salt” is mostly inferred by “sodium” labelled on the NIP; **5)** Understanding of NRV%, the percentage that the content of a nutrient (sodium here) in 100 g of the food takes up out of the recommended daily intake; **6)** Use of the sodium label, defined as checking the NIP most of time when shopping, with the purpose of buying the less salt-laden pre-packaged food; and **7)** Knowledge, attitude, and behavior of salt intake. In this study, six grams per day of salt was considered the recommended amount as per the Dietary Guidelines for Chinese Residents (2016) in China [20]. Based on series of expert panel discussion, we designed three single-choice questions to evaluate attitudes towards salt intake (awareness of healthy salt levels in food), which was then transformed into an attitude score (Table 1). Salt reduction behavior by asking: “Are you trying to control salt intake during daily life?” which refers to how people reduce salt intake cooking at home, eating out or buying pre-packaged food. (See Additional file 1: Questionnaire for detail).

**Table 1:** Questions, answers and rating principal of participants’ attitude towards salt intake

No.	Question	Answers	Score ^a^
1	Do you believe that excessive salt intake does harm to your health?	Strongly believe	3
		Believe	2
		Maybe	1
		No	0
2	Do you believe that reduction in salt intake can help to lower blood pressure?	Strongly believe	3
		Believe	2
		Maybe	1
		No	0
3	Do you think it is necessary to control salt intake in pre-packaged foods?	Very Necessary	3
		Necessary	1.5
		Not Necessary or Indifferent	0

^a^ The score of attitude towards salt intake was summarized and translated into 100 points for each participant

### Statistical analysis

Mean ± Standard Deviation (SD) and 95% Confidence Intervals (95% CI) were used to describe continuous variables, and percentage and 95% CI were used to express categorical variables. The t-test and chi-square test were used to compare continuous variables and categorical variables among different subgroups, respectively. Binary logistic regression analyses by forward stepwise (Likelihood Ratio) method (the entry and removal probabilities for stepwise are 0.05 and 0.1, respectively) were applied in order to assess the factors associated with the participants’ awareness, understanding, and use of current sodium labels. Demographic, existing health conditions, knowledge and awareness of healthy amount of salt-intake were controlled in the multivariate analyses. SPSS software (Statistical Package for the Social Sciences, Version18.0) was used.

## Results

### Characteristics of the participants

Of the 750 people approached, all the 380 community residents completed the investigation. Of the 370 shoppers, 50 chose not to participate, and 12 more did not fill out the entire questionnaire. The final analysis included 380 (55.2%) community residents and 308 (44.8%) supermarket shoppers. Among the 688 successful respondents, the average age was 39.9 ± 15.0, most (78.8%) were female, more than half (55.7%) had junior college or above educational level and around half were suffering from hypertension (51.6%) or cardiovascular disease (40.8%). More details about the characteristics of the participants in community residents and supermarket shoppers are shown in Table 2.

**Table 2:** Characteristics of the participants (*N* = 688)

	Total population (N = 688)	Community residents (*n* = 380)	Supermarket shoppers (*n* = 308)
Sex (Male), %	31.2	26.3**	37.3
Age (year), mean ± SD	39.9 ± 15.0	42.5 ± 16.1**	36.7 ± 12.7
Educational level, %			
Junior high school or below	18.6	13.9**	24.4
High or technical school	25.7	23.9	27.9
Junior college	16.3	16.1	16.6
Undergraduate	29.8	35.5	22.7
Graduate or above	9.6	10.5	8.4
Household monthly income (Yuan) per capita, %			
≤ 5000	59.7	57.4**	62.7
50,001–10,000	26.3	31.6	19.8
10,0001–20,000	10.6	7.6	14.3
≥ 20,0001	3.3	3.4	3.2
BMI (kg/m^2^), mean ± SD	23.1 ± 3.2	23.2 ± 3.2	23.1 ± 3.2
Existing health problems, %			
Hypertension	51.6	55.0*	47.4
Cardiovascular disease	40.8	43.4	37.7
Chronic kidney disease	12.4	13.4	11.0
Knowledge, attitude and behavior towards salt intake			
Know recommended daily salt intake, %	69.3	72.6*	65.3
Score of attitude towards salt intake, mean ± SD ^a^	66.1 ± 19.9	68.2 ± 19.5**	63.6 ± 20.1
Salt reduction behavior, % ^b^	67.9	73.9**	60.4

^*^ *p* < 0.05; ^**^ *p* < 0.01 when compared between community residents and supermarket shoppers

^a^ The attitude towards salt intake was assessed by calculating the score of the participants’ answers to three questions and translated into 100 points

^b^ The behavior of usually controlling the salt intake in daily life

### Knowledge, attitude and behavior on salt intake

69.3% knew the recommended amount of daily salt intake (6 g/day). The majority (91.3%) of the participants believed excessive salt intake to be harmful to their health; 76.0% believed that a reduction in salt intake could help to lower a person’s blood pressure; and the average score of attitude towards salt intake was 66.1 out of a perfect 100 points. 67.9% had the behavior of reducing salt intake in daily life.

### Awareness of sodium labels

The survey found that 19.5% (95% CI: 16.5%–22.4%) of participants were aware that sodium was required to be labeled on the NIP of pre-packaged foods. Table 3 shows the awareness of the sodium label in total and subgroups of the study population. In Table 4, the logistic regression analysis indicated that younger people, those with cardiovascular disease and those with a higher level of education and awareness of salt in the diet made people read the labels on food. Table 4 provides more details of the relationship between the taking notice of the sodium content listed on sodium labels and a person’s age, educational level and history of cardiovascular disease. Participants with hypertension were not more aware of the sodium label in either univariate or multivariate analyses (*P* > 0.05).

**Table 3:** The awareness, understanding, and use of sodium labels among participants (%): univariate analysis

Factors	N	Awareness ^a^	Understanding ^b^	Use ^c^
Overall	688	19.5	52.3	12.6
Sex				
Male	215	15.8	47.9	8.4^*^
Female	473	21.1	54.3	14.6
Age				
18–25	117	33.3^**^	42.7	9.4
26–35	228	22.8	54.4	12.7
36–45	116	20.7	55.2	14.7
46–59	149	7.4	55.7	13.4
≥ 60	78	10.3	50.0	12.8
Source of participants				
Community	380	21.3	56.8^**^	16.8^**^
Supermarket	308	17.2	46.8	7.5
Educational level				
Junior high school or below	128	5.5^**^	35.2^**^	6.3^*^
High or technical school	177	12.4	39.0	11.9
Junior college	112	20.5	59.8	20.5
Undergraduate	205	27.8	63.9	13.2
Graduate or above	66	37.9	72.7	12.1
Household monthly income per capita				
≤ 5000	411	13.6^**^	47.0^**^	9.2^*^
5001–10,000	181	28.2	62.4	17.7
10,001–20,000	73	26.0	54.8	16.4
≥ 20,0001	23	34.8	60.9	21.7
BMI				
< 24	441	21.3	53.1	10.4
24–27	194	15.5	51.0	17.0
≥ 28	53	18.9	50.9	15.1
Hypertension				
Yes	355	21.1	56.1*	14.1
No	333	17.7	48.3	11.1
Cardiovascular Disease				
Yes	281	26.7^**^	59.1^**^	16.0^*^
No	407	14.5	47.7	10.3
Chronic Kidney Disease				
Yes	85	29.4^*^	63.5^*^	18.8
No	603	18.1	50.7	11.8
Awareness of recommended daily salt intake				
Yes	477	21.2	59.7^**^	14.3
No	211	15.6	35.5	9.0
Score of attitude towards salt intake ^d^				
Higher	304	22.7	57.9^**^	20.7^**^
Lower	384	16.9	47.9	6.3
Salt reduction behavior ^e^				
Yes	467	19.7	58.0^**^	15.4^**^
No	221	19.0	40.3	6.8

^a^ Proportion of participants aware of sodium label on nutrition information panel

^b^ Proportion of participants who understood the relationship between sodium and salt

^c^ Proportion of participants who read or checked the sodium label most of time while shopping

^d^ The score of participants towards salt intake. “Higher” score was defined as above the average, and “Lower” score was defined as below the average

^e^ The behavior of usually controlling the salt intake in daily life

^*^ *p* < 0.05; ^**^ *p* < 0.01, the chi-squared test was used to compare the differences among subgroups

**Table 4:** Associated factors: awareness, understanding and use of sodium labels. Results of logistic analysis by a stepwise method

Final model ^a^	β	SE ^b^	*P* value	Odds Ratio	95% CI of OR	
					Lower	Upper
Awareness of sodium label						
Age	−0.501	0.100	< 0.001	0.606	0.498	0.737
Educational level	0.416	0.088	< 0.001	1.516	1.276	1.801
Cardiovascular Disease	0.704	0.210	0.001	2.022	1.339	3.052
Score of attitude towards salt intake ^c^	0.020	0.006	< 0.001	1.020	1.009	1.031
Constant	−3.111	0.517	< 0.001	0.045	─	─
Understanding of sodium label ^d^						
Educational level	0.436	0.065	< 0.001	1.547	1.361	1.757
Awareness of recommended daily salt intake	0.937	0.183	< 0.001	2.553	1.784	3.653
Salt reduction behavior ^e^	0.512	0.178	0.004	1.668	1.177	2.365
Constant	−2.149	0.267	< 0.001	0.117	─	─
The use of sodium label ^f^						
Income level	0.355	0.143	0.013	1.426	1.078	1.886
Score of attitude towards salt intake ^c^	0.037	0.007	< 0.001	1.038	1.025	1.052
Source of participants	−0.870	0.268	0.001	0.419	0.248	0.708
Awareness of sodium label	0.553	0.272	0.042	1.739	1.020	2.964
Constant	−4.522	0.727	< 0.001	0.011	─	─

^a^ Sex, age, source of participants, educational level, household monthly income per capita, BMI (kg/m^2^), existing health problems, awareness of recommended daily salt intake, score of attitude towards salt intake, and salt reduction behavior were included in the model at the first step

^b^: Standard error

^c^: The score of participants towards salt intake, translated into 100 points

^d^: Knowing the relationship between salt and sodium

^e^: The behavior of usually controlling the salt intake in daily life

^f^: Reading or checking nutrition label most of time when shopping

### Understanding sodium labels

About half (52.3%, 95% CI: 48.6%–56.1%) of the participants understood the relationship between sodium and salt, but only 5.5% (95% CI: 3.8%–7.2%) of all participants understood the meaning of NRV%. Table 3 shows in more detail, the understanding of the relationship between sodium and salt among different groups of people. Because the understanding of NRV% was too low to conduct an analysis of what affected people’s awareness of NIPs, we only conducted logistic regression analysis to examine the factors that affect the understanding of the relationship between sodium and salt. In Table 4, the analysis indicated that participants who had a higher educational level, were more aware of the recommended daily salt intake, and managed their diet better as well as the amount of salt they consumed in food each day, were more likely to understand the relationship between sodium and salt.

### Use of the sodium label

Of all the participants, 12.6% (95% CI: 10.2%–15.1%) reported that they frequently or often (most of time) read or checked the sodium label when shopping. Univariate analysis indicates that the following people were more likely to effectively read and act on NIPs: females, community residents (versus supermarket shoppers), those with a higher educational level people with a higher monthly household income per capita plus if people had cardiovascular disease (Table 3). Multivariate analysis shows the similar results (Table 4).

## Discussion

In urban Beijing, our survey found that most participants had a good basic knowledge of salt and its harmful effects on health. Over two thirds of participants were aware of the recommended daily salt intake in China and the majority of them recognized the harm of a diet high in salt, believing that reducing salt intake is beneficial. However, only half of the urban adults know the relationship between “sodium” and “salt”, very few people (5.5%) understood the meaning of NRV%, less than one-fifth (19.5%) of them were aware of the sodium label, and only 12.6% used (read or checked) the sodium label while purchasing pre-packaged foods. Those with a higher income and higher education levels had an increased awareness of and understanding of sodium labels. Considering that this was conducted in urban Beijing where people have nearly the highest income and education levels in China, the level of the awareness, understanding of health diets and sodium labels might be less elsewhere in the country.

The high awareness (91.3%) of about the harmfulness of excessive salt intake found in this study reflects the success of Government’s “salt” reduction advocacy. The results showed that, participants with hypertension were no more aware of the “sodium” label than those without hypertension. This might be largely due to the low awareness of the relationship between ‘sodium’ and ‘salt’ as well as the low level of awareness of NRV% and sodium.

In our study, only about one-half (52.3%) of the participants reported knowing the relationship between salt and sodium. A similar study conducted in Japan in 2012 concluded that few people (only 13.3%) understood the correlation of 1000 mg sodium in grams of salt (2.50–2.60 g) [18]. This suggests that the current label of showing salt by sodium content may not be effective in informing consumers of the salt content in food. It would therefore be prudent to update the design and content of the NIP to show salt rather than sodium in order to improve awareness and understanding. This is also suggested by the CODEX Guidelines on Nutrition Labelling [21], and some countries such as UK, have mandatorily required to use “salt” rather than “sodium” on the NIP because the term “salt” is more readily comprehensible by consumers than “sodium” [22].

The reported use of the sodium label in our study was significantly lower than a similar study conducted in New York City (50.0% ± 2.2%) [19]. Moreover, in our logistic regression analysis, although we found that educational levels meant a higher awareness of the sodium label and better understanding of the relationship between salt and sodium, it did not transfer to the use of the sodium label when other factors were controlled. This means that although people with relatively high education levels were equipped with some basic knowledge and understanding of the sodium label, this knowledge did not result in them reading the NIP when shopping. This may be partially due to that health is not always the top driver of food choice.

Compared to the traditional NIP on the back of pack, front-of pack (FoP) labelling may be another effective way to convey sodium or salt information to the public. FoP labelling has been adopted by some countries to provide clearer information on nutrition information of food. In June 2013, the UK introduced a voluntary hybrid front-of-pack system that displays traffic-light colors to indicate whether the salt level is ‘low’ (green), ‘medium’ (amber) or ‘high’ (red), to help consumers make ‘at a glance’ healthier choices [23]. FoP labelling has been proven in various studies to be easier to understand and more effective for consumers in helping them to choose healthier foods [17, 18, 24, 25]. In addition, FoP labelling is believed to encourage food manufactures to reformulate their foods. In the 1980s, Finland introduced legislation to require food products containing a high level of salt to carry a high salt warning [15]. As a result, food companies reformulated their products and some high salt products disappeared completely from many shops.

Assisting consumers to choose lower-salt foods should not be limited to nutrition labelling itself. Smartphone applications by various research groups to help the public choose healthier food. For instance, The George Institute for Global Health launched an ‘app’ called FoodSwitch in 2012 in Australia and more recently in China, which aimed to help consumers choose healthier foods. Consumers scan the barcode on the package of the food and the nutritional information is presented in traffic light colour-coded format. In addition, healthier alternatives will be listed underneath the NIP of the scanned food [26]. Furthermore, the ‘app’ also includes a SaltSwitch filter which allows consumers to choose foods lower in salt [27]. A recent study has shown that SaltSwitch helpful consumers choose low-salt foods [26]. Considering the increasing coverage of smartphone (58% of Chinese people have smartphones) an app might be successful in helping people reduce their salt intake.

However, evidence has shown that the implementation of education and awareness-raising interventions alone are unlikely to be adequate in reducing population salt intake to the recommended levels [28]. New methods of health promotion on salt reduction should also be considered, for example, advocacy in schools. A study conducted in Northern China showed that integrating salt reduction into routine education for primary school children to be very successful. [29].

Several limitations existed in our study. Firstly, the survey was conducted in central districts of Beijing, where the level of awareness of sodium labels is higher than average. Secondly, as the study sample was not population-representative the results may not be representative of the whole country. Thirdly, nearly all the questions we designed were single or multiple choice. This may skew results due to a limited choice for answers on the questionnaire.

## Conclusions

Our survey showed that the participants had a good understanding of the harmful effects of salt, but the awareness and use of sodium labels was very low in Beijing. Few people understand what NRV% means on food packaging. There may be less awareness of salt and NIPs nationwide. Efforts should be taken to educate the public to understand and use the NIP better and design clearer ways of displaying such information, such as front-of pack (FoP) labelling or health-related smartphone applications to improve health and help people make better food choices.

## Additional file

Additional file 1: Questionnaire. (DOCX 66 kb)

## Abbreviations

CI: Confidence Intervals
FoP: Front-of pack
NIP: Nutrition information panel
NRV%: Nutrient reference value
SD: Mean ± Standard Deviation
WHO: World Health Organization
