# Supplementary material for: Awareness, understanding and use of sodium information labelled on pre-packaged food in Beijing:a cross-sectional study
Source: BMC Public Health. 2018 Apr 17;18:509. doi: 10.1186/s12889-018-5396-7 (PMC5905172; doi:10.1186/s12889-018-5396-7)
Supplement: Supplementary file 1 — Questionnaire. (DOCX 66 kb) [file 12889_2018_5396_MOESM1_ESM.docx]

# Additional file 1: Questionnaire

A survey of understanding and use of nutritional labels among urban residents in Beijing

Unit: Peking University Health Science Center

No:

Note: There is only one choice if not specified.

## 1. Basic information

1. Please confirm your age:

①18～25 ②26～35 ③36～45 ④46～59 ⑤≥60

1. Please confirm your gender: ①Male ②Female
2. Please confirm your height: cm
3. Please confirm your weight: kg
4. Please confirm your educational level:

①Junior high school or below ②High school ③Technical secondary school

④Undergraduate ⑤Graduate or above

1. Please confirm your household monthly income per capital (RMB) :

①≤2,000 ②2,001-5,000 ③5,001-10,000 ④10,001-20,000 ⑤>20,000

1. Please confirm whether you have the following existing health problem (s) (choose one or more)?

①Hypertension ②Diabetes ③Cardiovascular disease ④Dyslipidemia

⑤Fatty liver ⑥Chronic kidney disease ⑦Food allergy ⑨None

## 2. Related knowledge and attitude

1. Are you concerned about your health?

①Very concerned ②Somewhat concerned ③Not very concerned

④Not too much ⑤Not at all

1. Do you think you know well about balanced diet？

①Very well ②Generally

③Not too much ④Not at all

1. How much of a threat do you think poor diet is to health?

①Very much ②Relatively big

③Not too much ④Not at all

1. Do you control the intake of oils and fats?

①Yes ②No

1. Why don’t you control the intake of oils and fats (choose one or more)?

①The foods with less oils and fats are not delicious. ②I do not cook, so I can’t control. ③There is no harm with greater intake. ④My intake is already minimal.

⑤I don’t gain weight even if I eat more. ⑥I don’t care.

1. Where does energy mainly come from (choose at least one):

①Proteins ②Fats ③Carbohydrates ④Sodium ⑨I don't know

1. Excessive energy intake may lead to (choose one or more):

①Diabetes ②Mental illness ③Hepatitis ④Obesity ⑨I don't know

1. What foods are the main sources of high quality protein (choose one or more)?

①Milk ②Fishes ③Peanuts ④Fruits and vegetables ⑤Legumes ⑨I don't know

1. Which of the following ingredients cannot be absorbed by human body but beneficial to health?

①Sucrose ②Starch ③Dietary fiber ④Glucose ⑨I don't know

1. What’s the recommended daily salt intake limit for Chinese adults?

①2g ②6g ③9g ④12g ⑨I don't know

1. Are you trying to control salt intake during daily life?

①Yes ②No

1. Why don’t you control the intake of salt (choose one or more)?

① The foods with less salt are not delicious. ② I do not cook, so I can’t control.

③ There is no harm with greater intake. ④ My intake is already minimal.

⑤ Eating less salt is not conducive to health. ⑥ I don’t care.

## 3. Understanding and use of nutritional labels

1. How often do you purchase pre-packaged foods?

①Almost every day②3-5 times per week③1-2 times per week ④Less than 1 time per week

1. What’s the labeling requirement of China's current nutrition labeling of pre-packaged foods?

①Mandatory ②Voluntary ⑨I don't know

1. Do you know what nutritional ingredients must be labeled on pre-packaged foods in China?

①No ②Not sure ③Yes

1. What nutritional ingredients must be labeled on pre-packaged foods besides energy and protein in China?^[[1]](#footnote-1)^

①Fat, carbohydrate, salt ②Fat, carbohydrate, sodium ③Trans-fat, sugar, sodium ④Saturated fat, sugar, sodium ⑤Saturated fat, dietary fiber, salt ⑨I don't know

1. What is the most common labeling mode of protein in nutritional labels?

①xx mg per 100mg ②xx g per serve

③xx g per 100g/ml ④xx g per 50g/ml ⑨I don't know

1. Do you know the meaning of “NRV%”?

①No, never heard of it. ②Heard of it, but it's not very clear to me. ③Yes, I Know.

1. Do you know the meaning of “营养素参考值百分比” (Chinese translation of “NRV%”)?

①No, never heard of it. ②Heard of it, but it's not very clear to me. ③Yes, I Know.

1. What can be inferred by "sodium” in nutritional labels?

①Mineral ②Carbohydrate ③Salt ④Fat ⑨I don't know

1. Is the current nutrition labeling helpful for you to choose pre-packaged foods?

①Very helpful ②Somewhat helpful ③Not too much ④Not at all

1. If the current nutrition labeling is not helpful to you, the most important reason is:

①I choose pre-packaged foods according to experience or knowledge.

②I don't know what the nutrition labeling is or don't know its function.

③The nutrition labeling is difficult to understand, so I do not know how to use it.

④I don’t trust the information labeled in the nutritional labels.

⑤The font in the nutritional labels are too small to see clearly.

⑥The color of the nutritional information panel is similar to the background in the package so that I can’t see clearly.

⑦I understand the nutritional labels, but it’s difficult to make any reference (specify )

⑧Other reason:

1. Do you have a habit of referring to the nutritional information panel (NIP) with the purpose of choosing healthier pre-packaged food while shopping?

①No ②Yes

1. Do you have the habit most of the time while shopping?

①No ②Yes

1. Which ingredient(s) labeled in nutritional information panel do you usually refer to?

①Never ②Energy ③Protein ④Carbohydrate ⑤Fat ⑥Sodium ⑦Other, specify

1. Have you ever learned the knowledge of food nutrition labeling?

①No ②Not sure ③Yes

1. What channels do you prefer to get nutrition labeling information (choose 3 at most)?

①Clearer and easier to understand food nutritional labels.

②Mass media (radio, television, newspapers and other news media).

③New media (Internet, WeChat, micro-blog).

④The small media (billboards, brochures or advertising slogans).

⑤The professionals (medical staff, nutritionist).

⑥Family members, colleagues or friends.

⑦ Others, specify .

1. Have you ever seen this label while shopping (Appendix 2-Figure A)?

① Yes, often. ②Yes, sometimes. ③No

1. If you have seen this label, is it helpful for you to choose pre-packaged foods?

①Very helpful ②Not too much ③Not at all

1. Which of the following labels are easier to understand and do you prefer (Appendix 2-Figure B-E)?

①Percentage of Guideline daily amount (GDA %) ②The traffic light label

③Health star rating system ④5-color ⑤Health logo

1. Do you want your favorite label you chose above to be labeled on all pre-packaged foods?

①Yes, very much. ②Yes.

③It does not matter. ④No, it’ s unnecessary, the current label is good.

1. What nutrients do you hope most to be added in the label?

①There is no need to add. ②I don't understand, so I can't choose. ③Saturated fat

④Trans-fat ⑤Dietary fiber ⑥Sugar ⑦Cholesterol ⑧Calcium ⑨Others, specify

1. If you have any other suggestions for improving the nutritional label, please explain:

.

This is the end of the questionnaire. Thank you for your participation!

1. The question 22 and 23 are one question in fact. The respondent would get full score (100 points) only if he chose “Yes” for question 22 and correct answer for question 23,otherwise he would get 0 point. [↑](#footnote-ref-1)
